# Mosquitoes on a plane: Disinsection will not stop the spread of vector-borne pathogens, a simulation study

**DOI:** 10.1371/journal.pntd.0005683

**Published:** 2017-07-03

**Authors:** Luis Mier-y-Teran-Romero, Andrew J. Tatem, Michael A. Johansson

**Affiliations:** 1 Dengue Branch, Division of Vector-Borne Diseases, Centers for Disease Control and Prevention, San Juan, PR; 2 WorldPop, Department of Geography and Environment, University of Southampton, Southampton, United Kingdom; 3 Flowminder Foundation, Stockholm, Sweden; 4 Center for Communicable Disease Dynamics, Harvard TH Chan School of Public Health, Boston, Massachusetts; Oxford University Clinical Research Unit, VIET NAM

## Abstract

Mosquito-borne diseases are increasingly being recognized as global threats, with increased air travel accelerating their occurrence in travelers and their spread to new locations. Since the early days of aviation, concern over the possible transportation of infected mosquitoes has led to recommendations to disinsect aircraft. Despite rare reports of mosquitoes, most likely transported on aircraft, infecting people far from endemics areas, it is unclear how important the role of incidentally transported mosquitoes is compared to the role of traveling humans. We used data for Pla*smodium falciparum* and dengue viruses to estimate the probability of introduction of these pathogens by mosquitoes and by humans via aircraft under ideal conditions. The probability of introduction of either pathogen by mosquitoes is low due to few mosquitoes being found on aircraft, low infection prevalence among mosquitoes, and high mortality. Even without disinsection, introduction via infected human travelers was far more likely than introduction by infected mosquitoes; more than 1000 times more likely for *P*. *falciparum* and more than 200 times more likely for dengue viruses. Even in the absence of disinsection and under the most favorable conditions, introduction of mosquito-borne pathogens via air travel is far more likely to occur as a result of an infected human travelling rather than the incidental transportation of infected mosquitoes. Thus, while disinsection may serve a role in preventing the spread of vector species and other invasive insects, it is unlikely to impact the spread of mosquito-borne pathogens.

## Author summary

Vector-borne diseases such as malaria, dengue, yellow fever, and Zika are global challenges to public health. International policies, such as the International Health Regulations, call for controlling mosquitoes on aircraft to prevent the introduction of mosquito-borne pathogens and invasive mosquito species. The research presented here used malaria and dengue data to estimate the likelihood of introduction of both pathogens via humans and mosquitoes travelling on airplanes. We found that the probability of introduction of either pathogen by mosquitoes is low due to few mosquitoes being found on aircraft, low infection prevalence among mosquitoes, and a short lifespan. Humans were hundreds of times more likely to spread pathogens via air travel, even in the absence of any mosquito control. Therefore, policies designed to prevent the transportation of infected mosquitoes on airplanes are unlikely to prevent the spread of vector-borne diseases.

## Introduction

Mosquito-borne diseases such as malaria [1] and dengue [2] are major causes of morbidity and mortality globally. While these pathogens are endemic only in tropical and subtropical environments, modern air travel has broken down traditional geographic barriers to the extent that infected humans and mosquitoes may quickly travel anywhere in the world [3, 4]. Infected travelers of either variety pose a risk of introducing pathogens to areas where the environment is suitable but the pathogen is not present, leading to local outbreaks. Furthermore, in areas where the pathogen does exist, introductions may result in the replacement of a local strain with a newly introduced one, perhaps with different pathogenicity or drug resistance profiles.

Despite the recognized and realized risk that travelling humans and mosquitoes accidentally transported on aircraft present for the spread of vector-borne diseases [3], there remains a great deal of uncertainty about how best to minimize the spread of pathogens via these two hosts. One option is disinsection, “the procedure whereby health measures are taken to control or kill the insect vectors of human diseases present in baggage, cargo, containers, conveyances, goods and postal parcels.”[5] The broad implementation of such measures has been in place for many decades [6] and is specifically spelled out in the International Health Regulations [5], which state that, “Every conveyance leaving a point of entry situated in an area where vector control is recommended should be disinsected and kept free of vectors.” Effective disinsection could reduce the risk of spread of both specific mosquito species and pathogens [3]. However, specifically for pathogen introduction, it is unclear how important the role of incidentally transported mosquitoes is compared to the role of traveling humans.

Introduction of a pathogen by either a human or a mosquito depends on a sequence of stochastic events. Starting in the source location, there is a probability that a human or mosquito is infected by the pathogen and a probability that a human or mosquito travels to another location. Upon arrival, there is some probability of transmission. For the infected human, this depends on being found by a competent mosquito, being fed upon during the infectious period, having that mosquito first survive the extrinsic incubation period and then transmit the pathogen to another person. For a transported infected mosquito, only the latter part of this sequence is required; the mosquito must survive the extrinsic incubation period and transmit the pathogen to a person before dying.

Here we developed branching process models to compare the risk associated with traveling mosquitoes and traveling humans. Branching process models can be used to explicitly formulate chains of discrete random events such as those described above [7, 8]. In this framework, each stochastic step is first characterized individually and then the sequential series of events is analyzed using well-developed mathematical methods. Here, we are interested in a single outcome, introduced autochthonous transmission, which could result from two alternative pathways, introduction by an infected human or by an infected mosquito.

We focused on two mosquito-borne pathogens of global importance, the malaria parasite *P*. *falciparum* and dengue viruses. These pathogens differ in important ways, they are transmitted by different vectors (Anopheles and Aedes mosquitoes, respectively) and have different human infection dynamics (multi-stage, long-term infection or single stage acute infection, respectively). To assess the worst-case scenario for potential introduction, we focused on highly endemic areas where transmission of each pathogen is near optimal and formulated a stochastic description of each step of the composite process using published literature to estimate the key parameters. We then estimated probabilities of introduction via each pathway and assessed the sensitivity of those outcomes to assumptions incorporated into the model.

## Materials and methods

We used a branching process framework to model *P*. *falciparum* and dengue virus introduction via air travel by either infected mosquitoes or humans. In brief, a branching process is composed of a sequence of stochastic steps, each one taking discrete input from the preceding step and producing discrete stochastic output [7, 8]. The pathogen introduction pathway via mosquitoes included the following steps:

1. There must be at least one mosquito on the aircraft. We modeled the number of mosquitoes as a Poisson process with mean *λ*_*M*_, the average number of mosquitoes found on an aircraft.
2. There must be at least one infected mosquito. We modeled this step as a Bernoulli process where each mosquito has probability *p*_*IM*_ of being infected with the pathogen.
3. An infected mosquito must transmit the pathogen to at least one human. We modeled this step as a Poisson process, with a mean of *R*_*0MH*_, the average number of humans infected per infected mosquito.

Introduction via humans includes the following steps:

1. There must be at least one human on the aircraft. We modeled this step as a Poisson process with mean *λ*_*H*_, the average number of humans found on an aircraft.
2. There must be at least one infected human. We modeled this step as a Bernoulli process where each human has a probability *p*_*IH*_ of being infected with a pathogen.
3. An infected human must arrive and transmit the pathogen to at least one mosquito. We modeled this step as a Poisson process, with a mean of *R*_*0HM*_, the average number of mosquitoes infected per infected human.
4. An infected mosquito must transmit the pathogen to at least one human. We modeled this step as a Poisson process, with a mean of *R*_*0MH*_, the average number of humans infected per infected mosquito. This step is identical to the final step for introduction by a mosquito as the key outcome in both pathways is a human infection.

To capture the full sequence of events in each pathway, we sequentially combined the probability generating functions for each individual step to build a generating function for each complete sequence of events (S1 Text). With these composite functions, we calculated the probability that at least one human transmission event occurred at the destination. This approach enabled the robust consideration of uncertainty and variability in the Poisson and Bernoulli parameters described above; each parameter is considered as a distribution of possible values rather than a fixed value. The key data and assumptions are described below, while further detail is presented in the Supporting Information (S1 Text). Lastly, we assessed the sensitivity of the branching process model results to parameter assumptions for each individual step by estimating the relative change in introduction probabilities per unit relative change in each parameter (S1 Text).

### Travel

**Fig 1:**
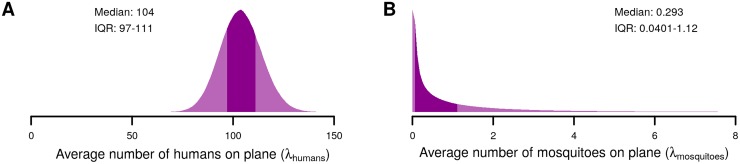
Distributions of the parameters for the average number of humans (A) and mosquitoes (B) on aircraft. The dark shaded areas in each distribution indicate the interquartile range. The median and interquartile range (IQR) of each distribution is shown in the corresponding panel.

The average number of human passengers on flights globally was 104 in 2015 according to the International Civil Aviation Organization, which we assumed to be Poisson distributed (Fig 1A) (http://www.icao.int/annual-report-2015/Documents/Appendix_1_en.pdf). Approximately 0.91 mosquitoes (95%CI: 0.00009–5.3) were found per aircraft on average across 17 studies of 559,579 aircraft from 1931 to 1999 [9–25] (Fig 1B). Because of the variety of aircraft, time periods, locations, objectives, methods, and data reported in these studies, we made the conservative assumption that all mosquitoes were competent vectors, female, and alive, and therefore capable of being infected and transmitting each pathogen. This scenario should therefore overestimate vector mosquito transportation by aircraft.

### Plasmodium falciparum

**Fig 2:**
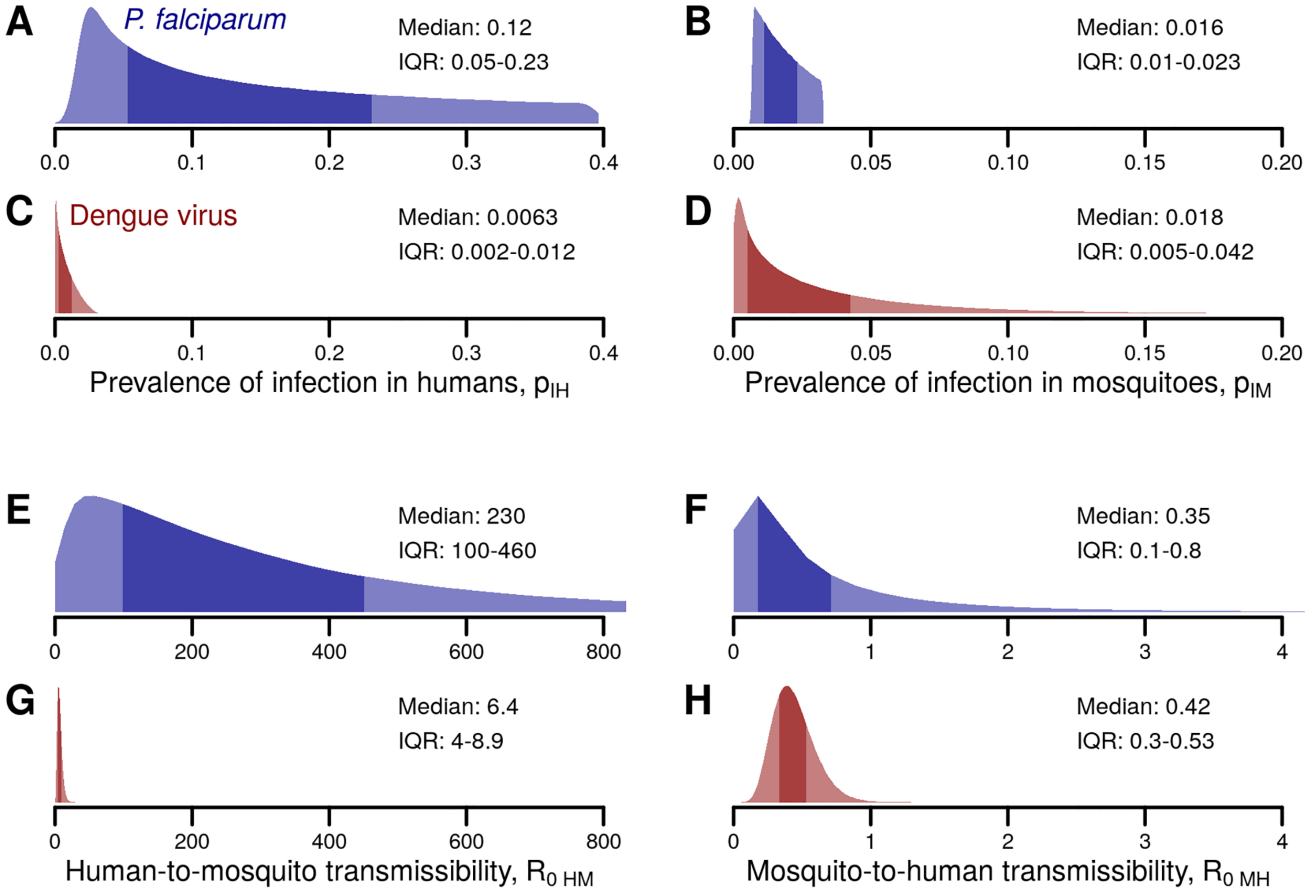
Distributions of parameters in the branching process for *P*. *falciparum* and dengue virus introduction. The prevalence of infection in humans and mosquitoes is characterized by Bernoulli distributions with parameters p_IH_ (A and C) and p_IM_ (B and D), respectively. Transmissibility for humans to mosquitoes and mosquitoes to humans are characterized by Poisson distributions with parameters R_0HM_ (E and G) and R_0MH_ (F and H), respectively. The dark shaded areas in each distribution indicate the interquartile range. Each panel provides the median and the interquartile range (IQR) of the corresponding distribution.

Estimates of the prevalence in humans were based on over 3,000 surveys of 2–10 year olds [26]. The prevalence of *P*. *falciparum* reported in these surveys ranged from 0% to almost 100%, with a mean of 23% (Fig 2A). Fewer data were available for mosquito populations, but the estimated prevalence of sporozoites in mosquitoes across 41 studies ranged from 0.01% to 7.6% with a mean of 2.3% [27] (Fig 2B). Because our focus was the relative probability of introduction by each pathway, we reduced extreme variability by sampling only from the interquartile range of the distributions of *p*_*IH*_ and *p*_*IM*_. Finally, to account for correlation between human and mosquito prevalence when comparing the two pathways, the samples were split into deciles and randomly paired within each decile, such that samples with relatively high human infection prevalence also had relatively high vector infection prevalence.

*R*_*0HM*_ was calculated as the product of the mosquito density *r* (mean: 31 mosquitoes per person [27]), mosquito biting rate *b* (above), the human-to-mosquito transmissibility *p*_*HM*_ (mean: 0.16 [1]), and the duration of infectiousness *D* (mean: 205 days [28]) (Fig 2E):

$$R_{0HM}=rbp_{HM}D.$$

*R*_*0MH*_ was calculated using the classic formulation [29]:

$$R_{0MH}=\frac{bp_{MH}}{\mu }e^{-\mu \cdot EIP},$$

accounting for the mosquito biting rate *b* (mean: 0.4 bites per day [27]), the mosquito-to-human transmissibility *p*_*MH*_ (mean: 0.55 [1], the mosquito mortality rate *μ* (mean: 0.13 per day [27]), and the length of the extrinsic incubation period, *EIP* (mean: 10.9 days [27]) (Fig 2F).

### Dengue virus

Estimates of the prevalence of DENV infection in humans were based on twenty seroprevalence studies (primarily in children) in hyperendemic locations across the globe [30–49]. The mean yearly infection rate ranged from 2% to 90%, averaging approximately 23%. To estimate the average daily prevalence of incubating and infectious humans, we sampled the duration of infection as the sum of the intrinsic incubation period (*IIP*, mean: 5.9 days [50]) and the adjusted infectious period (*D*, mean: 5.0 days, see below) discounted by the overlap (*O*, approximately 1 day [51]), and multiplied yearly incidence by (*IIP* + *D*–*O*)/365. The mean prevalence of DENV infection in humans was approximately 0.08% (Fig 2C). Mosquito infection rates were estimated from 13 studies in areas with ongoing dengue outbreaks that provided either direct measurements of infection rates, minimum infection rates through pooled samples or indirect measurements of infection rates via maximum likelihood estimates of pooled samples [52–64]. The mean prevalence of DENV infection in mosquitoes was 3% (Fig 2D). As for malaria, we sampled from the interquartile ranges of each distribution and stratified sampled human and vector prevalence by deciles.

*R*_*0MH*_ and *R*_*0HM*_ for DENV (Fig 2G and 2H) were estimated as for malaria (above) under the assumption that temperature was approximately 30°C, i.e. conducive to efficient dengue transmission [65]. The mean mosquito biting rate *b* was 0.7 bites per day [66], the mean mosquito-to-human transmissibility *p*_*MH*_ was 0.5, the mean mosquito mortality rate *μ* was 0.21 per day [67], and the mean length of the extrinsic incubation period, *EIP* was 6.5 days [50], and the mean mosquito density *r* was 2 mosquitoes per person [68]. To obtain the dengue parameter *R*_*0HM*_, we do not estimate the parameters *p*_*HM*_ and *D* independently, as was done for malaria. Rather, we obtain them in aggregate as the ‘Human Total Infectiousness’ (*HTI*), by integrating a logistic function of the human-to-mosquito transmissibility over the course of infection [69].

## Results

**Fig 3:**
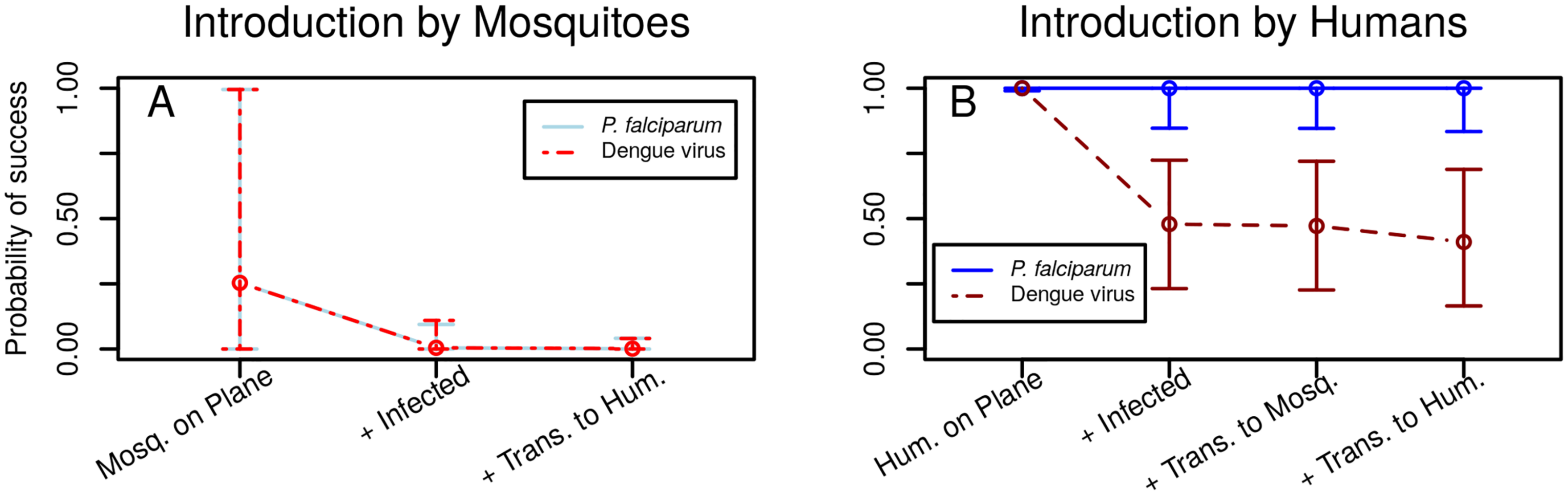
Step by step probabilities of success for each introduction pathway. For each sequential step in the mosquito (A) and human (B) introduction processes, we calculated the median (points) and 95% Credible Interval (vertical lines) for the probability of at least one event occurring. For example, the median probability that at least one human is on an aircraft is approximately 1.0 while the median probability that there is at least one human on an aircraft AND who is infected with a dengue virus is approximately 0.5. Note that for the introduction pathway via infected mosquitoes, the probabilities of success at each step (as well as the 95% CI) are nearly identical for both malaria and dengue.

The stepwise probabilities of *P*. *falciparum* or dengue virus introduction were quantitatively nearly identical for the mosquito pathway (Fig 3A). The probability of a mosquito being on an aircraft was generally low but variable (median: 0.25, 95% Credible Interval (CI): 9×10^−5^–0.995). The median probability of at least one mosquito on board being infected was much lower, less than 0.005 for both pathogens and the median probability for an infected mosquito traveling and subsequently transmitting to a human was less than 0.002. Humans, in contrast, were always present on aircraft and had high probabilities of completing each subsequent step of the introduction pathway: at least one traveler being infected, at least one instance of transmission to a mosquito, and at least one instance of transmission to a human (Fig 3B). For traveling humans, the median probability of a local transmission event was approximately 1.0 and 0.4 for *P*. *falciparum* and dengue viruses, respectively.

**Fig 4:**
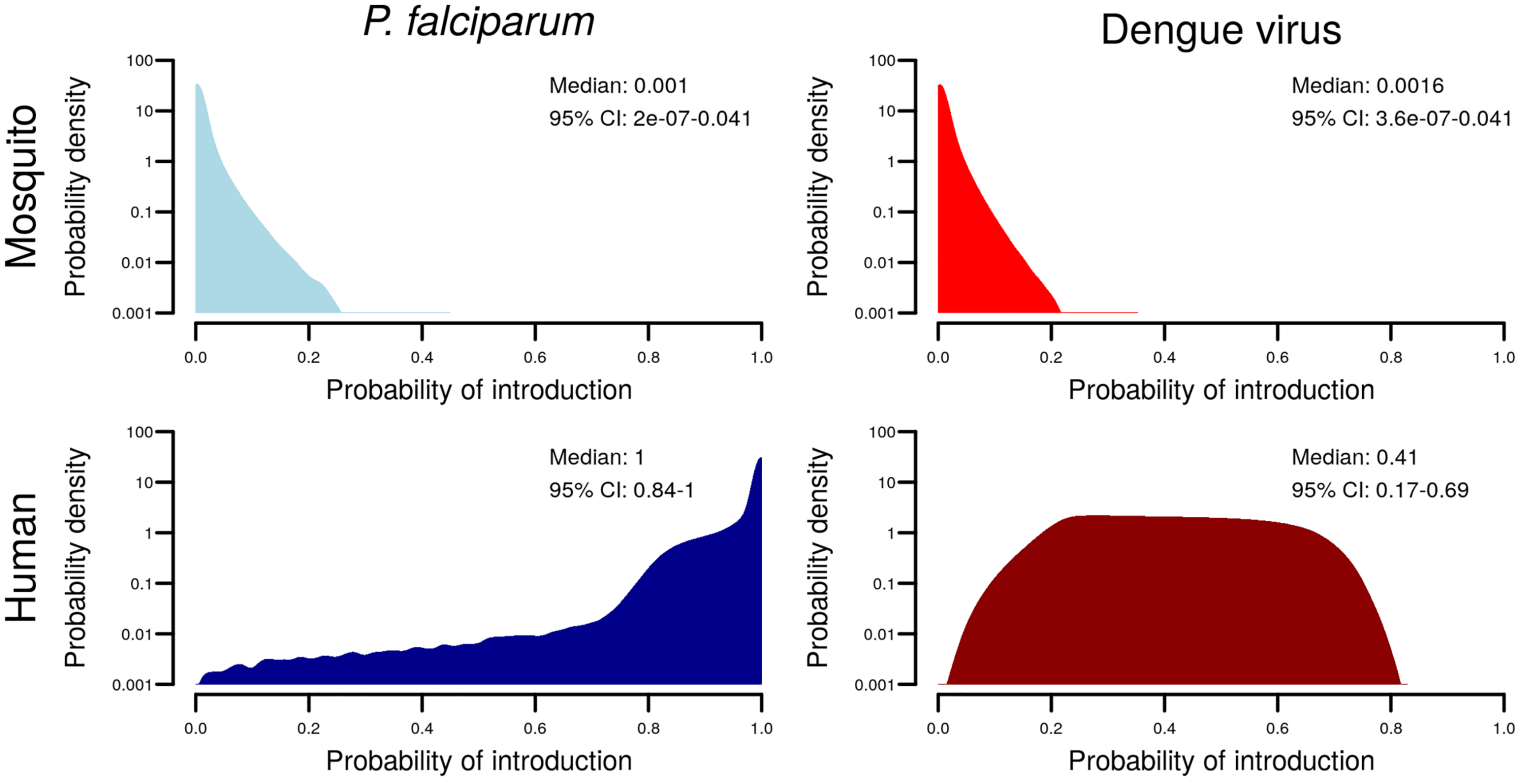
Distributions for the probability of introduction by each pathway. The density (log scale) for the probability of introduction via each pathway across 1 million simulations for *P*. *falciparum* (left column) and dengue virus (right column) and for the two pathways of introduction: infected mosquitoes (top row) and infected humans (bottom row). Each panel provides the median and 95% credible interval.

Overall, the median probability that a single aircraft traveling from an endemic area to another highly suitable area would lead to autochthonous human transmission of *P*. *falciparum* due to incidental transportation of mosquitoes was 0.001 (95% CI: 0.000–0.041). In contrast, the median probability of introduction by infected human travelers was 1.00 (95% CI: 0.84–1.00). For dengue viruses, the probabilities were 0.002 (95% CI: 0.00–0.04) for mosquitoes and 0.41 (95% CI: 0.17–0.69) for humans (Fig 4). The average odds of introduction by humans versus mosquitoes was 1,000:1 (95% CI: 500:1–1,800:1) for malaria and 240:1 (95% CI: 150:1–370:1) for dengue. For details on how these were obtained, see S1 Text.

The difference in introduction probabilities between the mosquito and human pathways starts with different probabilities of travel; multiple humans are found on most aircraft, while mosquitoes are relatively rare. For malaria, infection was more prevalent in humans (mean: 0.15) than in mosquitoes (mean: 0.02), so that the median estimated numbers of malaria *infected* humans and mosquitoes on an aircraft were 12 (95% CI: 2–40) and 0.005 (95% CI: ~1.4×10^−6^–0.1), respectively. The estimated probability of having an infected human onboard was approximately 200 (95% CI: 10–1×10^6^) times higher than the probability of an infected mosquito being onboard. For dengue, the probability of being infected was higher for mosquitoes in the midst of an outbreak (mean: 0.03) compared to average human prevalence over time periods encompassing outbreaks (mean: 0.007). Nevertheless, the difference in frequency of travel still translates to the probability of infected human travelers being approximately 93 (95% CI: 5–4.0×10^5^) times higher than the probability of infected mosquitoes being on an aircraft.

**Fig 5:**
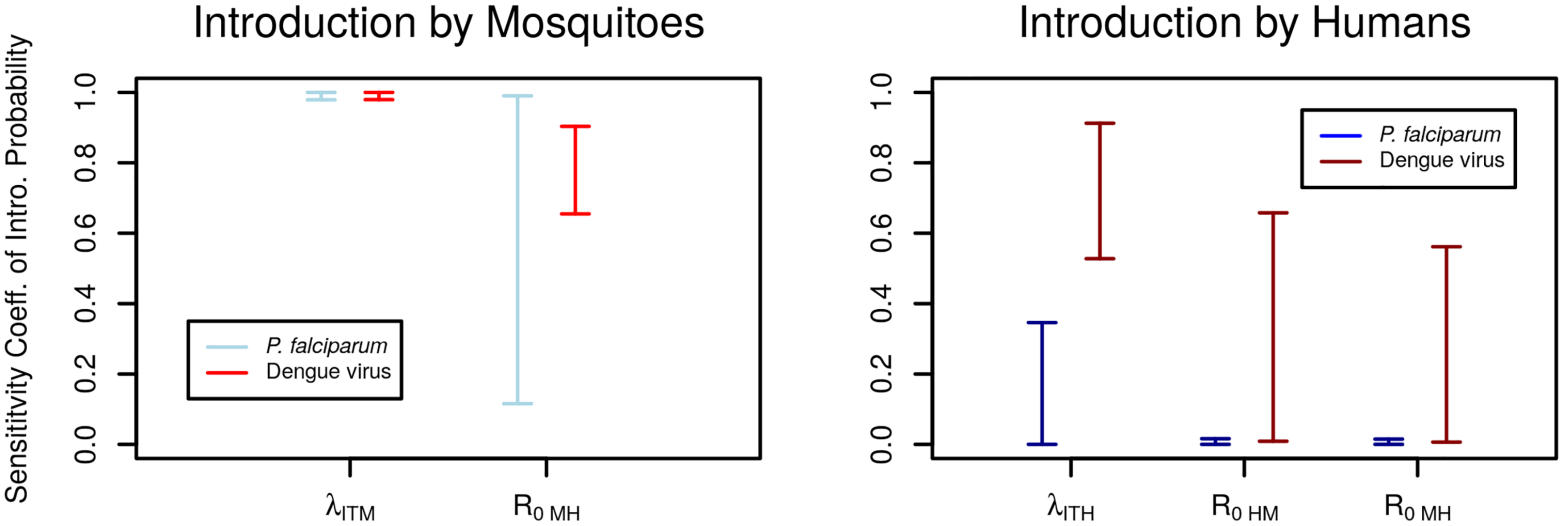
Sensitivity of introduction pathways to each individual step. Sensitivity coefficients indicate the percent change in the probability of introduction given a 1% change in each individual parameter (S1 Text). The probability of travel and the prevalence of infection are combined in the parameters λ_ITM_ and λ_ITH_.

We assessed the importance of each pathway-specific sub-component by estimating sensitivity coefficients (Fig 5). Sensitivity related to the prevalence of infection is intrinsically linked to the number of mosquitoes or humans on an aircraft because both contribute to the mean number of infected individuals onboard, λ_ITM_ and λ_ITH_, respectively. The mosquito pathway was highly sensitive to λ_ITM_ for both pathogens, a 1% change in either the number of mosquitoes or the prevalence of infection in mosquitoes resulted in a change of approximately 1% in the probability of introduction. The importance of R_0MH_ was lower, with a mean change of approximately 0.8% in introduction probability per 1% change in R_0MH_. Sensitivity for the human introduction pathway varied between malaria and dengue: *P*. *falciparum* introduction was mostly insensitive to parameter changes as introduction was highly probable across the parameter space while dengue showed some sensitivity, especially to the number of humans on an aircraft and the prevalence of infection (combined in λ_ITH_).

## Discussion

Mosquito-borne diseases such as malaria, dengue, yellow fever, chikungunya, and Zika are all endemic in tropical or subtropical areas. These diseases have also been documented among travelers and pose a transmission risk in areas where the pathogen is absent but the relevant mosquito vector is present, as has seen with chikungunya and Zika viruses in recent years. This risk is also a serious concern for areas where interventions are being targeted to prevent the invasion of drug resistant pathogens or to eliminate a pathogen altogether, such as current efforts to curb malaria [70, 71]. We assessed the relative risk of vector-borne pathogen introduction by infected humans and infected mosquitoes aboard aircraft, specifically estimating the probability of introduction of *P*. *falciparum* and dengue viruses. To clarify the relative risks of the two alternative pathways we focused on a situation that would favor introduction, in which the origin of air travel was assumed to be a highly endemic location and the destination was assumed to be equally suitable for transmission but nevertheless free of the pathogen. Introduction via infected human travelers was far more likely than introduction via infected mosquitoes; more than 1000 times more likely for *P*. *falciparum* and more than 200 times more likely for dengue viruses.

The low probability of introduction by mosquitoes stems from three key components. First, mosquitoes are rarely found on aircraft; the majority of aircraft from the 17 surveys had no mosquitoes on them and the highest number of vector species reported on a single aircraft was 17 *Anopheles gambiae* mosquitoes (possibly including both males and females) [13]. Second, if mosquitoes do make it onto aircraft, they are unlikely to be infected; the estimated infection prevalence of infection in mosquitoes was generally well below 5% even under the optimal conditions assessed here. The importation of *P*. *falciparum* or dengue virus via mosquitoes only becomes likely in the event that both of these rare conditions occur together. Finally, for a human to become infected at the destination, an imported, infected mosquito must survive long enough to complete the incubation period and feed on another human. Even in the highly endemic settings considered here, most infected mosquitoes do not survive long enough to transmit the pathogen.

The introduction probabilities differed between the two pathogens primarily because of the difference in infectious period (approximately 205 days for *P*. *falciparum* versus approximately 5 days for dengue viruses), which imparts increased human infection prevalence and increased human to mosquito transmission. Despite this difference and an estimate of higher prevalence in mosquitoes than humans, the relative risk for human introduction was still 200 times higher than for introduction by mosquitoes. This supports the generalizability of the finding that the frequency of travel and the differential transmissibility (human-to-mosquito being higher than mosquito-to-human) drive the difference in introduction risk.

The estimates provided here reflect a worst-case scenario in which prevalence is high in the source location and transmission is highly likely in the destination location. This choice was made to explicitly focus on the relative probability of introduction via human or mosquitoes as opposed to the absolute probability. In more realistic situations, the risk of introduction by either humans or mosquitoes is likely to be much smaller due to a number of factors influencing the association between infection risk and travel likelihood. For example, infection risk may be lower among traveling humans compared to the general population because they may have different exposure risk (e.g. if staying in an air-conditioned hotel) or different infection risk (e.g. adults are more likely to be immune to DENV infection). To assess how this might change the relative probability of introduction, we simulated a 90% reduction in human infection prevalence. Even under this condition, human travelers were more likely to introduce both pathogens than accidentally transported mosquitoes (S1 Text). Moreover, airports are often far from rural areas with high *P*. *falciparum* prevalence, so the prevalence of infection in both humans and mosquitoes on aircraft is likely much lower than estimated here. And efforts to limit transmission further reduce this risk [72]. The frequency of vector mosquitoes being accidentally transported on aircraft was also likely overestimated as we used average numbers of all mosquitoes regardless of species, sex, and viability (many were reported dead in the studies). These factors all suggest that we have overestimated the risk of introduction, especially by mosquitoes. However, the differences in transmissibility and human travel likelihood would change little, so the risk posed by travelling humans remains substantially higher than the risk posed by the accidental transportation of mosquitoes.

Recommendations for disinsection targeted at mosquitoes of public health importance focus on safety, effectiveness of the disinsection process, and the prevention of the introduction of invasive mosquito species and invasive pathogens via infected mosquitoes [3, 73]. The safety of passengers, crew, and the physical aircraft are key challenges not addressed here [73]. Effectiveness of the disinsection process itself is an additional concern; methods and implementation of disinsection vary, few insecticides are approved for use on aircraft, and many mosquitoes are resistant to some or all of those insecticides [73]. Safe and effective disinsection may help reduce the threat of vector mosquito invasion via aircraft to areas where those mosquitoes do not already exist [73], though some key vector species (e.g. *Ae*. *aegypti)* are already widely distributed and introduction has most often been attributed to shipping [74–76]. The importation of vector mosquito species was not addressed further here as this work was limited to the risk of mosquito-borne pathogen introduction. This introduction can manifest as a single transmission event (e.g. airport malaria or airport dengue) or the more problematic initiation of local transmission. Our results show that even in the absence of disinsection and under the most favorable conditions, the probability of any transmission resulting from the introduction of an infected mosquito by aircraft is very low. Moreover, the risk of introduced transmission via human travelers is 2–3 orders of magnitude higher. Cases of reported airport malaria or dengue number in the single digits per year [3, 77, 78], supporting the assertion that while infected mosquitoes on aircraft may transmit pathogens, it is extremely rare. Meanwhile hundreds to thousands of infections in human travelers are reported each year [79–84] each of which provides a higher likelihood of initiating transmission where vectors are present.

Concern about the spread of vector-borne pathogens via mosquitoes on aircraft has existed almost as long as aircraft themselves [9]. That concern continues to grow with the drastic increase in air travel and the arbovirus pandemics of recent years. Vector-borne pathogens are named as such for their dependence on insect vectors to complete the transmission cycle. However, on an international scale, clearly vector-borne diseases spread via humans travelling on aircraft, rather than insects. Even with perfect disinsection, which is far from guaranteed, the likely impact of disinsection on the spread of vector borne disease is negligible as that spread is many times more likely to occur due to human travel.

## Supporting information

S1 Text Supporting information on models, parameters, and sensitivity analyses. (PDF)
