# Supplementary material for: Mosquitoes on a plane: Disinsection will not stop the spread of vector-borne pathogens, a simulation study
Source: PLoS Negl Trop Dis. 2017 Jul 3;11(7):e0005683. doi: 10.1371/journal.pntd.0005683 (PMC5510898; doi:10.1371/journal.pntd.0005683)
Supplement: S1 Text — (PDF) [file pntd.0005683.s001.pdf]

Supporting Information on models, parameters, and sensitivity analyses

Contents

1) **Branching process models**
2) **Parameter distribution estimation**
3) **Sensitivity analysis**
4) **Relative odds of introduction**
5) **Decreased likelihood of infection in human travelers**

1) Branching process models

A branching process is composed of a sequence of stochastic events where the result of each one becomes the input for the following step. For example, here the number of human passengers on board aircraft is distributed as a Poisson random variable with parameter λ_{humans}. Given the stochastic outcome of this first step –the number of human passengers on the aircraft– the second step determines how many of the passengers are infected if each one of them has an independent probability of being infected p_{IH}. These stochastic events can be described and analyzed via their probability generating functions [*1, 2*]. The probability generating function for the number of traveling human passengers on the aircraft is:

$$g_{TH}(s) = exp\big(\lambda_H(s-1)\big).$$

The probability generating function describing the infection of a single human individual is:

$$g_{IH}(s) = 1 - p_{IH} + p_{IH} \cdot s.$$

The probability generating function of the composite process (the total number of *infected traveling humans*) is:

$$g_{ITH}(s) = g_{TH}\big(g_{IH}(s)\big) = exp\ \big(p_{IH}\lambda_H(s-1)\big).$$

Probability generating functions, $g(s)$, have the property that $g(0)$ is the probability that there are no offspring of the process and, thus, 1-$g(0)$ is the probability that there is at least one offspring. A composite probability generating function can do the same for an extended process that includes any number of stochastic steps. Hence, the probability of having at least *one* infected human on board the aircraft is:

$$1 - g_{ITH}(0) = 1 - exp\ (-p_{IH}\lambda_H).$$

Tables A and B show the generating functions for the steps leading to pathogen introduction via infected mosquitoes and infected human travelers, respectively, as well as the generating functions for the full chain in each instance. Given these composite functions for pathogen introduction, the probability of having at least one infected human at destination due to an infected onboard mosquito is:

$$1 - g_{TM}\Big(g_{\mathfrak{I}}\big(g_{MH}(0)\big)\Big).$$

While the analogous introduction probability via an infected human traveler is

$$1 - g_{TH}\Big(g_{IH}\Big(g_{HM}\big(g_{MH}(0)\big)\Big)\Big).$$

Table A: Generating functions for each of the step of introduction via mosquitoes on aircraft.

Step	Distribution	Generating Function
Number of Mosquitoes Onboard aircraft	Poisson	$g_{TM}(s) = exp\left(\lambda_M(s-1)\right)$
Infection of Individual Mosquito	Bernoulli	$g_{\mathfrak{I}}(s) = 1 - p_{\mathfrak{I}} + p_{\mathfrak{I}} \cdot s$
Transmission from Infected Mosquito to Human	Poisson	$g_{MH}(s) = exp\left(R_{0MH}(s-1)\right)$
Overall Process	-	$g_{TM}\left(g_{\mathfrak{I}}(g_{MH}(s))\right)$

Table B: Generating functions for each of the step of introduction via human travelers.

Step	Distribution	Generating Function
Number of Humans Onboard aircraft	Poisson	$g_{TH}(s) = exp\left(\lambda_H(s-1)\right)$
Infection of Individual Human	Bernoulli	$g_{IH}(s) = 1 - p_{IH} + p_{IH} \cdot s.$
Transmission from Infected Human to Mosquito	Poisson	$g_{HM}(s) = exp\left(R_{0HM}(s-1)\right)$
Transmission from Infected Mosquito to Human	Poisson	$g_{MH}(s) = exp\left(R_{0MH}(s-1)\right)$
Overall Process	-	$g_{TH}\left(g_{IH}\left(g_{HM}(g_{MH}(s))\right)\right)$

2) Parameter distribution estimation

For each parameter, we estimated a distribution representing variability and uncertainty. Here we describe how we estimated each distribution from available data and provide those distributions.

***Plasmodium falciparum* transmission**

Prevalence of P. falciparum in humans. For the parasite rate distribution around the globe, we performed an optimization over the two parameters of a Beta distribution in order to minimize:

$$Error = |q_1 - \hat{q}_1| + |q_3 - \hat{q}_3| + |\mu - \hat{\mu}|$$

where q_1, q_3 and μ are the values for first and third quantiles and mean, respectively, as reported by Guerra et al. [*3*] and $\hat{q}_1$, $\hat{q}_3$, and $\hat{\mu}$ are the estimated values from the Beta distribution.

Mosquito Mortality (μ). The mosquito mortality rate per gonotrophic cycle is

$$\mu_{gc} = \frac{1-M}{u}$$

where M is the probability of surviving one gonotrophic cycle and u is its duration [4]. The data for M and u are fit to a Beta distribution for μ_{gc} and then transformed to an instantaneous mortality rate μ via

$$\mu = -\log(1-\mu_{gc}).$$

All parameters related to *P. falciparum* transmission are shown in Table C.

Table C: Parameters related to *P. falciparum* transmission and their probability distributions.

**Quantity**	**Symbol**	**Mean and 95% CI**	**Distribution***
Prevalence of *P. falciparum* in humans	p_{IH}	0.24 (0.00, 0.90)	Beta(0.35, 1.11)
Prevalence of *P. falciparum* in mosquitoes	p_{IM}	0.02 (0.00, 0.09)	Beta(0.98, 40.8)
Mosquito density	r	32.8 (1.4, 112.1) per person	Gamma(1.2, 0.036)
Mosquito biting rate	b	0.30 (0.14, 0.53) per day	Beta(9.3, 30.5)
Human-to-mosquito transmissibility	p_{HM}	0.16 (0.14, 0.18)	Beta(306, 1900)
Mosquito-to-human transmissibility	p_{MH}	0.55 (0.47, 0.63)	Beta(82, 68)
Human Infectious Period	D	211 (185, 238) days	Gamma(242, 1.15)
Mosquito mortality per gonotrophic cycle	μ_{gc}	0.12 (0.03, 0.25)	Beta(3.9, 28.1)
Extrinsic Incubation Period	EIP	10.9 (8.4, 13.8) days	Gamma(63.4, 5.8)

*Distribution parameters given for Gamma distributions denote shape and rate parameters.

Dengue virus transmission

Human Infectious Period (D). For the four serotypes, we use the odds ratio (OR) for the probability of successful human to mosquito transmission and day of 50% infectivity to mosquitoes (t_{50}) from [5] (Table D) to fit a logistic function to the probability of human to mosquito transmission as a function of day of illness (t):

$$\pi_{HM}(t) = \frac{1}{1+e^{-\alpha_0-\alpha_1 t}},$$

where

$$\alpha_1 = \log(OR)$$
$$\alpha_0 = -\alpha_1 t_{50}.$$

Finally, we take D to be given as a Gamma fit to the length of time necessary for human to mosquito infectivity to drop to a threshold of p_{thrld} = 0.1 for the four serotypes and add one day to account for infectivity before illness onset:

$$D = 1 + \frac{1}{\alpha_1}\left(\log\left(\frac{p_{thrld}}{1-p_{thrld}}\right) - \alpha_0\right).$$

Table D. Odds ratios and time to 50% human to mosquito transmissibility from [5].

	OR (95% CI)	t_{50} (95% CI)
DENV1	0.23 (0.16, 0.35)	4.8 (4.5, 5.2)
DENV2	0.26 (0.19, 0.37)	4.1 (4, 4.5)
DENV3	0.35 (0.21, 0.57)	4.0 (3.5, 4.5)
DENV4	0.47 (0.27, 0.82)	2.9 (1.0, 3.5)

Human Total Infectiousness (HTI). To calculate R_{0HM} for dengue, we estimated total infectiousness rather than estimating p_{HM} and D independently, because the two components are linked and data exists to estimate their combined contribution [5].

Using $\pi_{HM}(t)$ as given in the previous section,

$$HTI = 1 + \int_0^\infty \pi_{HM}(t)dt,$$

we integrate the equation and calculate HTI using the values of α_0 and α_1 as indicated above:

$$HTI = 1 - \frac{1}{\alpha_1}\log(1 + e^{\alpha_0}).$$

All parameters related to dengue virus transmission are shown in Table E.

Table E: Parameters related to dengue virus transmission and their probability distributions.

Quantity	Symbol	Mean and 95% CI	Distribution*
Dengue incidence in humans	NA	0.25 (0.004, 0.74)	Beta(0.83, 2.53)
Intrinsic Incubation Period	*IIP*	5.9 (5.1, 6.7) days	Gamma(209, 35.4)
Human Infectious Period	*D*	7.0 (6.5, 7.5) days	Gamma(721, 103)
Prevalence of dengue viruses in mosquitoes	p_{IM}	0.02 (0.006, 0.04)	Beta(0.66, 21.17)
Mosquito density	*r*	2.0 (0.5, 4.4) per person	Gamma(4, 2)
Mosquito biting rate	*b*	0.7 (0.6, 0.8) per day	Beta(70, 3)
Human Total Infectiousness	*HTI*	5.0 (4.3, 5.7) days	Gamma(190, 38)
Mosquito mortality rate	μ	0.21 (0.18, 0.24) per day	Gamma(155, 736)
Mosquito–to-human transmissibility	p_{MH}	0.50 (0.29, 0.71)	Beta(10, 10)
Extrinsic Incubation Period	*EIP*	6.5 (4.6, 8.7) days	Gamma(39.2, 6.0)

* Parameters for the Gamma distributions denote shape and rate, respectively

3) Sensitivity analysis

We quantified the sensitivity of the introduction probabilities with respect to parameters using 'sensitivity coefficients' to describe how sensitive a certain quantity is with respect to small changes of one of its parameters, while other parameters remain fixed. The sensitivity coefficient SC_i of a quantity P with respect to its i-th parameter θ_i as:

$$SC_i = \frac{P}{\theta_i}\frac{\partial\theta_i}{\partial P}.$$

It measures the relative change in P per unit of relative change in the parameter θ. For example, a 1% increase in θ leads to an SC% change in P. Note that this sensitivity depends upon the value of all remaining parameters, as well as on the value of θ_i itself.

4) Relative odds of introduction

We calculated the odds of introduction via humans versus mosquitoes using for the estimated probability distribution. First, we matched deciles from human and vector distributions to capture the large-scale correlation that exists between the seroprevalence of both hosts. Within each decile, however, we paired individual probabilities. We then obtained the distribution of odds values by dividing the probability of introduction by humans ($p_{introHi}$) and by mosquitoes ($p_{introMi}$):

$$o_i = \frac{p_{introHi}}{p_{introMi}},$$

for *i = 1, 2,… N*. However, the many instances of infinitesimally small $p_{introMi}$, make the distribution of the o_i have a long tail which skews the calculation of means and medians towards very high values. To correct for this, we split the *N* values into *B* bins and calculate a median for each of the bins, resulting in *N/B* values med_j, for *j = 1, 2, … N/B* and a distribution representing uncertainty on the median estimated odds ratio.

5) Decreased likelihood of infection in human travelers

In this section, we explore the possibility that infection prevalence in humans may be inversely related to the probability of travel, for example if prevalence was highest in children or residents, but adults or visitors were more likely to travel. To simulate this possibility, we decreased the probability that a human traveler is infected by either malaria or dengue pathogens by 90% while leaving the infection rate of mosquitoes the same (Figure A). The median probability of *P. falciparum* introduction by infected human travelers is reduced to 0.70 (95% CI: 0.17–0.98). For dengue viruses, a 90% decrease in infection prevalence among human travelers reduced the median probability of introduction to 0.05 (95% CI: 0.02–0.11) (Figure A). Although the distribution of introduction probabilities via traveling mosquitoes remain as in the main text, they are shown here for comparison purposes. With the 90% reduction in infection prevalence among human travelers, the average odds of introduction by humans versus mosquitoes was 563:1 (95% CI: 376:1–672:1) for malaria and 31:1 (95% CI: 22:1–42:1) for dengue.

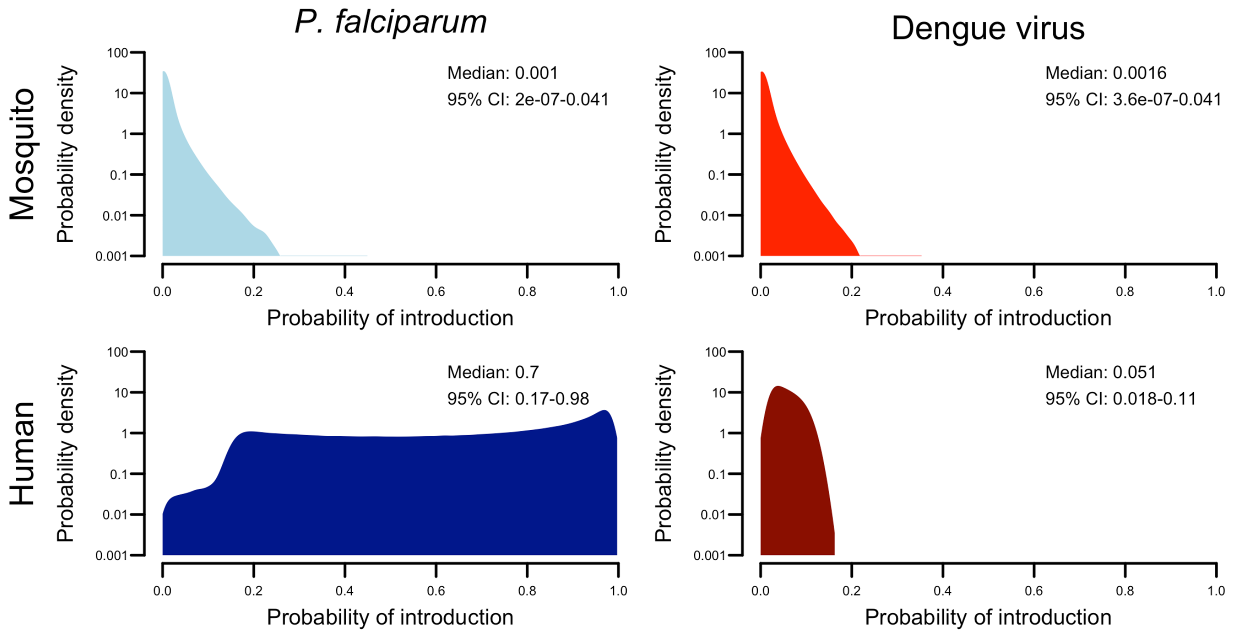

Figure A. Distributions for the probability of introduction by each pathway when the prevalence of infection in human travelers is reduced by 90%. The density (log scale) for the probability of introduction via each pathway across 1 million simulations for *P. falciparum* (left column) and dengue virus (right column) and for the two pathways of introduction: infected mosquitoes (top row) and infected humans (bottom row). Each panel provides the mean and 95% credible interval.

1. T. Harris, The theory of branching processes. Prentice-Hall Inc. (1963).
2. K. B. Athreya, A simple proof of a result of Kesten and Stigum on supercritical multitype Galton-Watson branching process. *The Annals of Mathematical Statistics*, 195-202 (1970).
3. C. A. Guerra *et al.*, The limits and intensity of Plasmodium falciparum transmission: implications for malaria control and elimination worldwide. *PLoS Med.* **5**, e38 (2008).
4. G. F. Killeen, A. Ross, T. Smith, Infectiousness of malaria-endemic human populations to vectors. *Am. J. Trop. Med. Hyg.* **75**, 38-45 (2006).
5. M. N. Nguyet *et al.*, Host and viral features of human dengue cases shape the population of infected and infectious Aedes aegypti mosquitoes. *Proc. Natl. Acad. Sci. U. S. A.* **110**, 9072-9077 (2013).
